# Validity and Reliability of Strategy Metrics to Assess Countermovement Jump Performance using the Newly Developed *My Jump Lab* Smartphone Application

**DOI:** 10.2478/hukin-2022-0098

**Published:** 2022-09-08

**Authors:** Chris Bishop, Paul Jarvis, Anthony Turner, Carlos Balsalobre-Fernandez

**Affiliations:** 1 London Sport Institute, Middlesex University, The Burroughs, London, UK; 2 Applied Biomechanics and Sport Technology Research Group, Autonomous University of Madrid, Madrid Spain

**Keywords:** app, biomechanics, jump strategy, profiling

## Abstract

The aim of the present study was to analyse the validity and reliability of the newly developed My Jump Lab smartphone app, which includes the option to calculate time to take-off and the reactive strength index modified (RSI_Mod – calculated as jump height divided by time to take-off), in addition to jump height. Twenty-seven postgraduate sport science students attended a single test session and performed three maximal effort countermovement jumps (CMJ) on twin force plates, whilst concurrently being filmed using the app. Results showed no significant differences in jump height between measurement methods (g = 0.00) or RSI_Mod (g = -0.49), although a significant difference was evident for time to take-off (g = 0.68). When a correction factor was applied to time to take-off data, no meaningful differences were evident (g = 0.00), which also had a knock-on effect for RSI_Mod (g = 0.10). Bland-Altman analysis showed near perfect levels of agreement for jump height with a bias estimate of 0.001 m, whilst time to take-off reported a bias estimate of 0.075 s initially and, 0.000 s once the correction factor was applied. For RSI_Mod, bias estimate was initially -0.048, and 0.006 once calculated with the corrected time to take-off data. Pearson’s r correlations were: 0.98 for jump height, 0.81 for time to take-off, and 0.85 for RSI_Mod. Based on the findings from the present study, and with the inclusion of the newly embedded correction factor, My Jump Lab can now be used as both a valid and reliable means of measuring time to take-off and RSI_Mod in the CMJ.

## Introduction

The countermovement jump (CMJ) is a common exercise used to assess lower body neuromuscular performance (Claudino et al., 2017). Not only does it represent one of the movement patterns frequently seen in various sports (e.g., basketball, volleyball, soccer, etc.) (Abdelkrim et al., 2007; Krol and Mynarski, 2012; Sheppard et al., 2009; Vaverka et al., 2013, 2016), but can also be considered one of the most time-efficient and useful tests in high-performance settings (Bishop et al., 2021a). When considering the methods available for assessing the CMJ, force plates are considered the gold standard as they enable the acquisition of force time-time data, and thus provide practitioners with the ability to quantify both the outcome measure (i.e., jump height), and also a range of strategy-based metrics (e.g., time to take-off, reactive strength index modified [RSI_Mod], impulse, etc.) with high levels of precision (Chavda et al., 2018; McMahon et al., 2018). Importantly, previous research has outlined that strategy metrics from the CMJ can be more sensitive at detecting true change compared to the outcome measure (Gathercole et al., 2015a, 2015b), as part of the continued monitoring process. Specific to the aims of this study, both RSI_Mod and time to take-off have been used in a number of empirical investigations and been shown to exhibit meaningful change between test sessions and time points (Bishop et al., 2022; Ebben et al., 2010; Suchomel et al., 2015).

Furthermore, a recent meta-analysis has highlighted the meaningful associations between reactive strength and independent athletic performance markers, such as linear and change of direction speed (Jarvis et al., 2021). As such, this strengthens the argument about the need to monitor more than jump height alone (Bishop et al., 2021a), and reinforces the value of force plates for practitioners.

Despite these benefits, not all practitioners have access to force plates because of their cost. Other methods often used include: the OptoJump (Bishop et al., 2022; Glatthorn et al., 2011), a jump mat (McMahon et al., 2016) and more recently, smartphone applications like My Jump 2 (Balsalobre-Fernandez et al., 2015). When considering data collection and subsequent analyses, devices such as optical measurement systems and contact mats are faster to use and "time" is always a factor in elite sports. However, despite the possible benefits, these devices are still expensive and there is no guarantee that practitioners with low budgets will have access to these two pieces of equipment either. In contrast, the My Jump 2 app costs £11.99 (UK pricing at the time the present study was conducted) and has been validated against a force plate for accurately assessing jump height using the flight time method of calculation (Balsalobre-Fernandez et al., 2015). For the CMJ, the app currently only offers data for jump height, with data related to strategy metrics (e.g., time to take-off and RSI_Mod) unavailable. However, given that time to take-off is defined as the time period where movement is initiated in the countermovement dip to the point where the athlete leaves the ground (Bishop et al., 2021b), this should be possible based on the same principles as previously validated for the acquisition of jump height, when using slow-motion video analysis in the app. Furthermore, RSI_Mod is calculated as jump height divided by time to take-off (Ebben et al., 2010; Suchomel et al., 2015), and therefore should also be possible to calculate. To date though, no study has aimed to quantify such metrics during the CMJ, using slow-motion video analysis. Thus, the main aim of this study was to analyse the validity and reliability of a new generation of the app (named: *My Jump Lab*) which includes a new option to calculate time to take-off and RSI_Mod. We hypothesised that a force plate would elicit significant differences in time to take-off values compared to the app, which would also result in meaningful differences for RSI_Mod.

## Methods

### Participants

Twenty-seven postgraduate sport science students (age: 26.26 ± 5.08 years; body height: 1.78 ± 0.06 m; body mass: 82.78 ± 11.73 kg) volunteered to participate in the present study. Inclusion criteria required all participants to be free from injury at the time of testing and have a minimum of one year’s strength and plyometric training experience. All subjects were required to provide written informed consent and this study was approved by the London Sport Institute research and ethics committee at Middlesex University (Application No: 19325).

### Design and Procedures

This study used a single session design based on the methods of the previously validated study for jump height using the *My Jump* app (Balsalobre-Fernandez et al., 2015). Each participant completed a standardised 10-min warm up consisting of 5 min of dynamic stretching. Specifically, this involved 1 set of 10 repetitions of: forward and lateral lunges, forward and lateral hip swings, bodyweight squats and the ‘world’s greatest stretch’. Participants then performed three practice trials of the CMJ, at their perceived maximal effort. For data collection, all jumps were performed on twin force plates (Hawkin Dynamics, Westbrook, ME, USA), whilst being concurrently filmed on a fourth generation iPad Pro 10 recording in slow motion at 240 frames per second. The iPad was mounted to a tripod at a height of 0.75 m at a distance of 3 m from the front of the force plates. This was deemed appropriate during pilot testing to capture the full frame of the participants throughout the ground contact phase of the jump, when recording in slow motion. In the original validation study, an iPhone camera focused on the feet only (Balsalobre-Fernandez et al., 2015). However, given the intention to calculate time to take-off in the present study, and how movement initiates at the hips or knees during the start of the descent of the CMJ (Rauch et al., 2020), camera placement was set to ensure capture of the full frame of the participants throughout the ground contact phase of the jump.

All the videos were then analysed using the *My Jump Lab* app installed on an iPhone 12 Pro running iOS 15.1. This app is the next generation of the previously validated *My Jump 2* app (Balsalobre-Fernandez et al., 2015) and uses the exact same algorithms for flight time and jump height detection. An update to the current *My Jump Lab* version (release 2.0) was specifically developed for this study using Xcode 13.1 for macOS Monterey 12.0.1 and the Swift 5 programming language with iOS 15 SDK (Apple Inc., USA). The update used the exact same video analysis framework from Apple to record and import slow-motion videos (AVFoundation, Apple Inc., USA) as the previously validated *My Jump 2* app, while including specific new features to allow the measurement of time to take-off, defined as the time between the first frame in which the start of the countermovement was visually detected, and the first frame in which both feet took-off the ground. It is worth noting for the reader that this particular version of the app doesn’t have any particular hardware requirement other than the ability to record videos at 240 frames per second, and can be installed on any device running iOS 13 or newer.

### Countermovement Jump Testing

All participants stepped onto twin force plates operating at 1000 Hz and were required to perform three maximal effort CMJ’s with 90-s rest intervals between jumps. Each trial was concurrently filmed using the aforementioned methods. Hands remained fixed on the hips and the legs were required to remain fully extended during the flight phase of the jump. Upon stepping onto the force plates, participants were required to stand motionless for 2 s so that bodyweight could be accurately determined (Perez-Castilla et al., 2018, 2021a, 2022). Jumps were initiated by asking participants to perform each jump for maximal height, with the countermovement phase self-selected to avoid any alterations in their preferred jump strategy. The first meaningful change in force was defined as any value greater than five standard deviations (SD) in body mass, as per the suggestions of Owen et al. (2014). Recorded metrics included: jump height, which was determined from the impulse-momentum method. This calculation method was chosen because it is the most accurate method of determining jump height (Chavda et al., 2018; McMahon et al., 2018; Owen et al., 2014) and the app has already been validated using the flight-time method in the initial study (Balsalobre-Fernandez et al., 2015). Additional metrics were: time to take-off (defined as the time period between the initiation of the countermovement to the moment of take-off) and RSI_Mod (calculated by dividing jump height by time to take-off). For time to take-off, it is important to note that on the force plate, the first point in time was determined in the same way as the first meaningful change in force (i.e., any value greater than five SD of body mass). However, when using the app, this was determined by visual inspection of when the first downward movement occurred at either the hips or knees, thus initiating the start of the unweighting phase of the jump.

### Statistical Analysis

All values were initially recorded as means ± SD in Microsoft Excel. Normality of the data was confirmed using the Kolmogorov Smirnov test (*p* > 0.05). Within-session reliability was computed for both measurement methods using the coefficient of variation (CV), calculated as: (SD/average)*100 and a two way random intraclass correlation coefficient (ICC 2,1) with absolute agreement and 95% confidence intervals (CI). CV values less than 10% were deemed acceptable (Cormack et al., 2008) and ICC values were interpreted in accordance with guidelines from Koo and Li (2016) where: > 0.90 = excellent, 0.75-0.90 = good, 0.50-0.74 = moderate, and < 0.50 = poor. Levels of agreement between the force plate and the app were determined from Bland-Altman plots with 95% upper and lower limits (Bland and Altman, 1986). In order to determine concurrent validity between measurement methods, Pearson’s correlation coefficients (*r*) were calculated, with 95% CI determined based on Fisher’s r-to-z transformation (Cumming, 2013), and accompanying standard error the estimate. In order to determine systematic bias between measurement methods, paired samples *t*-tests were conducted for each metric with statistical significance set at *p* < 0.05. In the eventuality that significant differences were evident between measurement methods, follow-up analysis was conducted using a correction factor (i.e., a linear regression equation where the dependent variable was the force plate data, and the independent variable was the My Jump Lab data) to determine whether such differences could be rectified. Finally, practical significance between the force plate and *My Jump Lab* was also determined using Hedges *g* effect sizes with 95% CI. These were interpreted in line with suggestions by Rhea (2004) relative to the “recreationally trained” sample in the present study: < 0.35 = trivial, 0.35–0.80 = small, 0.81–1.50 = moderate and > 1.50 = large.

## Results

All data were identified as normally distributed (*p* > 0.05). Table 1 shows mean ± SD data and reliability statistics for the force plate and app. This is presented in two different formats: 1) as raw data collected from each measurement method and, 2) with time to take-off and RSI_Mod once the correction factor has been applied to time to take-off. Both methods showed acceptable CV values for each metric (force plate ≤ 9.21%; app ≤ 7.29%) and ICC values were moderate to excellent for each metric using the force plate (0.66-0.97) and good to excellent when using the app (0.79-0.96).

**Table 1:** Mean ± standard deviation (SD), coefficient of variation (CV) with 95% confidence intervals (CI), intraclass correlation coefficient (ICC) with 95% CI for the force plate and My Jump Lab measures, with hedges g effect size (ES) data and 95% CI. Table presented both raw scores (top section) and with a correction factor applied for the My Jump Lab data (bottom section).

Jump		Force Plate			My Jump Lab		Hedges *g*
		
Variable	*Mean ± SD*	*CV (%)*	*ICC (95% CI)*	*Mean ± SD*	*CV (%)*	*ICC (95% CI)*	(95% CI)
	***Raw Scores***				***Raw Scores***		
Jump height (m)	0.37 ± 0.07	3.42 (2.63, 4.21)	0.97 (0.92, 0.99)	0.37 ± 0.08	4.10 (3.24, 4.96)	0.96 (0.90, 0.98)	0.00 (-0.55, 0.55)
TTTO (s)	0.79 ± 0.12	8.52 (6.51, 10.54)	0.66 (0.46, 0.81)	0.71 ± 0.11	6.14 (4.43, 7.85)	0.79 (0.64, 0.89)	**0.68 (0.12, 1.25)**
RSI_Mod	0.48 ± 0.09	9.21 (7.06, 11.36)	0.71 (0.52, 0.84)	0.53 ± 0.11	7.29 (5.48, 9.10)	0.87 (0.76, 0.94)	-0.49 (-1.04, 0.06)
	***Raw Scores***				***Correction Factor Applied***		
Jump height (m)	-	-	-	-	-	-	-
TTTO (s)	0.79 ± 0.12	8.52 (6.51, 10.54)	0.66 (0.46, 0.81)	0.79 ± 0.10	4.97 (3.82, 6.12)	0.80 (0.65, 0.90)	0.00 (-0.55, 0.55)
RSI_Mod	0.48 ± 0.09	9.21 (7.06, 11.36)	0.71 (0.52, 0.84)	0.47 ± 0.10	6.37 (5.01, 7.73)	0.90 (0.79, 0.95)	0.10 (-0.44, 0.65)

*Note: ES value in **bold** indicates statistically significant difference (p = 0.04) between force plate and My Jump Lab. m = metres; TTTO = time to take-off; s = seconds; RSI_Mod = reactive strength index modified*.

When assessing systematic bias for the raw data, jump height showed no differences between measurement methods (*g* = 0.00), time to take-off showed significantly lower values for *My Jump Lab* compared to the force plate (*g* = 0.68; *p* = 0.009), resulting in RSI_Mod showing non-significant small differences (*g* = -0.49). However, when the correction factor was applied both time to take-off, and as a result of this RSI_Mod, showed no differences between measurement methods (*g* = 0.00 and 0.10 respectively).

**Figure 1:**
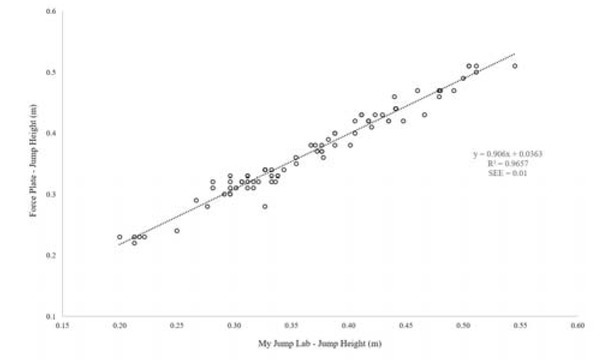
Scatter plot presenting a relationship between force plate and My Jump Lab methods for the calculation of jump height, including the correction factor and R^2^.

**Figure 2:**
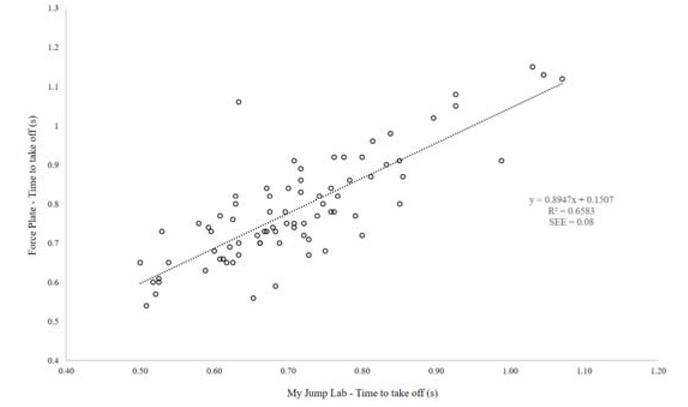
Scatter plot presenting a relationship between force plate and My Jump Lab methods for the calculation of time to take-off, including correction factor and R^2^.

**Figure 3:**
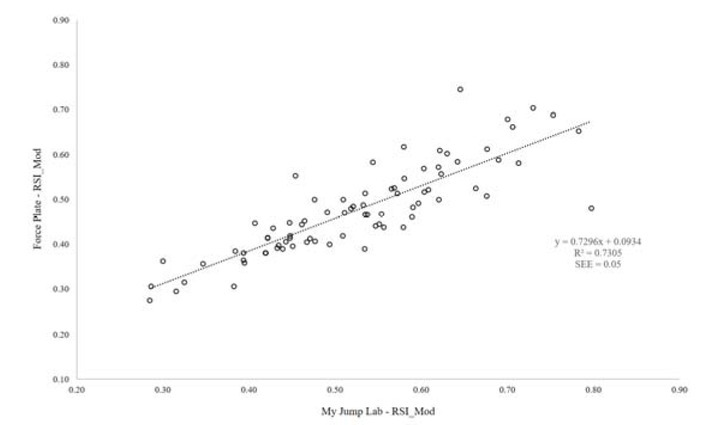
Scatter plot presenting a relationship between force plate and My Jump Lab methods for the calculation of RSI_Mod, including the correction factor and R^2^.

Figures 1-3 show scatter plot graphs presenting all trials for jump height (Figure 1), time to take-off (Figure 2) and RSI_Mod (Figure 3), with correction equations and *r^2^* values. Pearson’s *r* values were as follows: jump height (0.98 [0.97, 0.99]), time to take-off (0.81 [0.72, 0.88]), and RSI_Mod (0.85 [0.78, 0.90]). Specifically, time to take-off required the correction equation which was: y = 0.8947x + 0.1507, where y = the value for time to take-off measured from the force plate and x = the raw value for time to take-off computed in *My Jump Lab* (Figure 2). The reader should also note that the corrected RSI_Mod value was calculated by taking jump height and dividing it by the ‘corrected’ time to take-off value.

**Figure 4:**
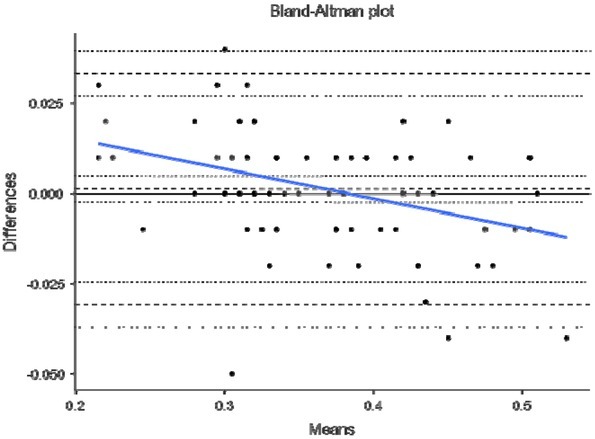
Bland-Altman plot depicting levels of agreement for the force plate and My Jump Lab for jump height, including bias estimate (0.001) and both lower (-0.031) and upper (0.033) limits of agreement.

**Figure 5:**
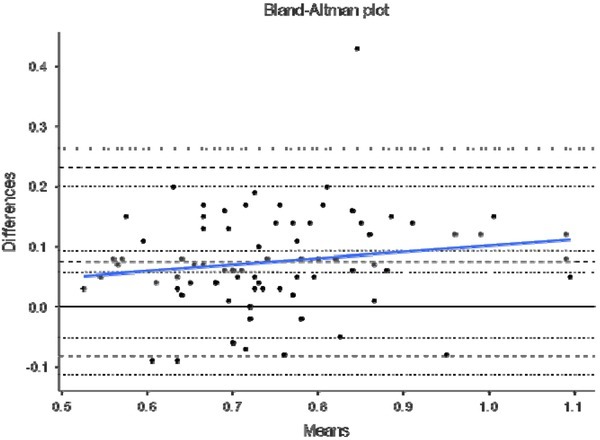
Bland-Altman plot depicting levels of agreement for the force plate and My Jump Lab for time to takeoff, including bias estimate (0.075) and both lower (-0.082) and upper (0.233) limits of agreement.

**Figure 6:**
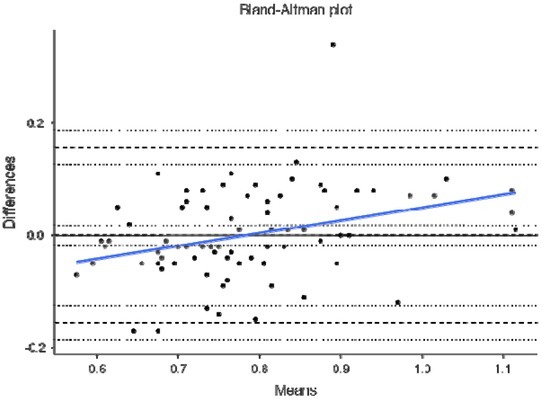
Bland-Altman plot depicting levels of agreement for the force plate and My Jump Lab (correction factor amended) for time to take-off, including bias estimate (0.000) and both lower (-0.156) and upper (0.157) limits of agreement.

**Figure 7:**
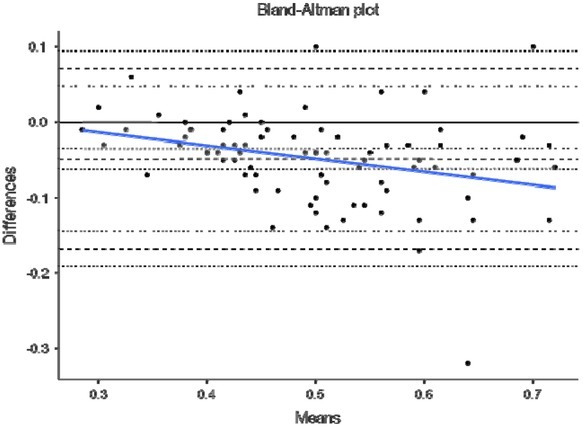
Bland-Altman plot depicting levels of agreement for the force plate and My Jump Lab for RSI_Mod, including bias estimate (-0.048) and both lower (-0.168) and upper (0.071) limits of agreement.

**Figure 8:**
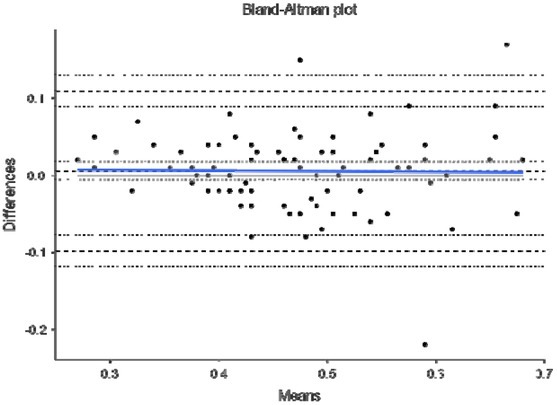
land-Altman plot depicting levels of agreement for the force plate and My Jump Lab for RSI_Mod (using correction factor amended data for TTTO), including bias estimate (0.006) and both lower (-0.098) and upper (0.110) limits of agreement.

Finally, Figures 4–8 show Bland-Altman plots for jump height (Figure 4), time to take-off raw data (Figure 5), time to take-off with correction factor (Figure 6), RSI_Mod raw data (Figure 7) and RSI_Mod with correction factor (Figure 8). Mean differences (bias estimates) for jump height were 0.001 m (i.e., 1 millimetre), showing almost perfect levels of agreement. Once bias estimates were applied to time to take-off (which showed an initial mean difference of 0.075 s), almost perfect levels of agreement were evident between the force plate and the app (0.000 s). Thus, when this was used to subsequently calculate RSI_Mod, this also resulted in near perfect levels of agreement between the two methods (0.006).

## Discussion

The aim of the present study was to analyse the validity and reliability of a new generation of the app (named: *My Jump Lab*) which includes a new option to calculate time to take-off and RSI_Mod. Results showed that jump height exhibited no differences between measurement methods, time to take-off showed significantly lower values for the app compared to the force plate, and RSI_Mod exhibited non-significant small differences. However, when the correction factor was applied to the time to takeoff metric, no differences were evident between measurement methods, which had the same effect on the subsequent RSI_Mod calculation. Both measurement methods showed acceptable variability for all metrics (CV < 10%), with the force plate exhibiting moderate to excellent ICC data (0.66–0.97) and the app showing good to excellent ICC data (0.79–0.96).

The most notable finding from the present study is that time to take-off exhibited significantly lower values than the force plate, when using the naked eye to determine the onset of movement during the CMJ. To understand why these differences occur, we must understand how movement is initiated and measured on a force plate. When performing a CMJ on a force plate, literature has highlighted that the first phase of movement to occur is the ‘unweighting phase’ (Chavda et al., 2018; McMahon et al., 2018; Owen et al., 2014).

This is where movement is initiated by the relaxation of the agonist muscles (Linthorne, 2001), which results in the start of flexion at the hips, knees and ankle joints. Furthermore, this initiation is identified when the first meaningful change in force surpasses a certain threshold, with a value greater than 5 x SD of body mass being suggested as a favourable method (Chavda et al., 2018; McMahon et al., 2018; Owen et al., 2014). This is because it allows for the same method to be used on all participants of different statures. On a force-time curve, this initial change in force would signify the beginning of the CMJ, which is evidently much more sensitive than what the naked eye can see when using the app. Further to this, the heightened sensitivity of force plates at detecting the first meaningful change in force, is exacerbated by the filtering process (Harry et al., 2022). Thus, it is our suggestion that practitioners do not manually calculate time to take-off, without the correction factor applied (which we have provided and is now fully embedded into *My Jump Lab* – meaning practitioners can use this metric straight away). This suggestion is supported by the reported bias in the raw data for this metric (0.075 s [lower -0.082 and upper 0.233 limits of agreement]; Figure 5) compared to when the correction factor is applied (0.000 s [lower - 0.156 and upper 0.157 limits of agreement]; Figure 6).

To follow on from this, RSI_Mod is also heavily affected by these data, noting it is calculated from jump height divided by time to take-off. Simply put, when using the raw data, because time to take-off produces a smaller number than the force plate (app = 0.71; force platform = 0.79 s), this causes RSI_Mod to be inflated when calculated in the app (app = 0.53; force plate = 0.48). Thus, the errors seen in time to take-off also have a pronounced effect (*g* = -0.49) on RSI_Mod when using the raw data. However, when the correction factor is applied to time to take-off, this new value can be used in the calculation of RSI_Mod, to produce an accurate value relative to the force plate (Table 1). This is again supported in the Bland-Altman plots which show bias of -0.048 [lower -0.168 and upper 0.071 limits of agreement] for the raw data (Figure 7) and 0.006 [lower -0.098 and upper 0.110 limits of agreement] when the correction factor is applied (Figure 8). Similar to our message for time to takeoff, the app now includes accurate RSI_Mod data, which are calculated from jump height divided by the correction factor transformed time to take-off data. An additional point to note for RSI_Mod is that it is a ratio (i.e., made up of two component parts). Recent literature has highlighted that ratios are often ‘noisier’ metrics; typically because when created, they pool two sources of variability together (Bishop et al., 2021a). Our data (Table 1) support this notion, with RSI_Mod exhibiting the largest CV values for any metric (although still less than 10%). Therefore, due to the heightened variability that often accompanies ratios, practitioners are advised to concurrently monitor the component parts (i.e., jump height and time to take-off) to ensure that they fully understand where any changes in RSI_Mod are being driven from.

An additional interesting discussion point relates to the almost identical values for jump height, despite the force plate calculating these values via the impulse-momentum method and the app using the flight-time method. During the initial validation study, Balsalobre-Fernandez et al. (2015) ensured the force plate calculated jump height via the flight-time method as well, to ensure that calculation methods were comparable. However, previous research has shown that net impulse is the key determinant of jump height (Ruddock et al., 2015), which provides the most accurate measure of take-off velocity, which in turn, determines the most accurate measure of jump height. Thus, the impulse-momentum method has been suggested to be the more favourable method of determining jump height from a force plate (Chavda et al., 2018; McMahon et al., 2018; Owen et al., 2014), especially when using bodyweight CMJ testing (Perez-Castilla et al., 2018). Despite the different calculation methods for jump height used in the present study, we actually believe this to be a strength of the study design – noting that the outcome of jump height was the same between measurement methods. This is further supported by the relationship between the two measurement methods for jump height (*r* = 0.98), and confidence interval thresholds of this value (0.97, 0.99).

Our final aim was to determine the reliability of these new metrics compared to the force plate. Relative reliability was worse for the time to take-off metric, with the app actually showing slightly better reliability than the force plate (app: ICC = 0.79; force plate: ICC = 0.66). Furthermore, all CV values were < 10% which can be deemed acceptable (Cormack et al., 2008; Turner et al., 2015), with RSI_Mod showing the largest variability of the metrics. Interestingly, the app actually showed a lower CV value than the force plate for RSI_Mod (app = 7.29%; force plate = 9.21%), with this difference increased further once the correction factor was applied in the app (6.37%). As such, practitioners can be confident that these new metrics provide reliable data when using *My Jump Lab*.

A couple of limitations of the present study should be noted. Firstly, only a single test session was employed for the primary purpose of this validation study. Future research should aim to conduct test-retest study designs using these newly validated metrics, to determine between-session reliability and minimal detectable change values. Secondly, we utilised sport science students and future research should also aim to determine the usability of these new metrics in elite athlete populations, given previous literature has highlighted the vast array of jump strategies that can be employed during the CMJ (Bishop et al., 2021a; Cormack et al., 2008; Gathercole et al., 2015a, 2015b). In summary though, practitioners can be confident that the *My Jump Lab* provides reliable and accurate data, relative to a force plate for jump height, time to take-off and RSI_Mod; which provides a more in-depth understanding of jump outcome and strategy during ongoing monitoring processes. As a final point of consideration, recent literature has highlighted the importance of linking metrics together, so that the interpretation of data is easier for practitioners (Bishop et al., 2021a). This is supported in new empirical research which has shown that the resultant RSI_Mod value is influenced by the level of depth during the countermovement (Perez-Castilla et al., 2021b). Thus, future research on the *My Jump Lab*, could investigate whether countermovement depth could be integrated into the app, offering practitioners even more information about CMJ strategy.

The findings from this study show that once the correction factor has been applied to the metric of time to take-off, it presents almost identical data to those of a force plate, which subsequently provides accurate RSI_Mod data as well. This enables practitioners to concurrently measure the outcome measure and jump strategy metrics using the newly developed *My Jump Lab* smartphone app.
